# Maternal sleep and small for gestational age infants in the Japan Environment and Children’s Study: a cohort study

**DOI:** 10.1186/s13104-017-2675-9

**Published:** 2017-08-11

**Authors:** Seiichi Morokuma, Mototsugu Shimokawa, Kiyoko Kato, Masafumi Sanefuji, Eiji Shibata, Mayumi Tsuji, Ayako Senju, Toshihiro Kawamoto, Koichi Kusuhara, Japan Environment & Children’s Study Group, Hirohisa Saito, Reiko Kishi, Nobuo Yaegashi, Koichi Hashimoto, Chisato Mori, Fumiki Hirahara, Zentaro Yamagata, Hidekuni Inadera, Michihiro Kamijima, Ikuo Konishi, Hiroyasu Iso, Masayuki Shima, Toshihide Ogawa, Narufumi Suganuma, Koichi Kusuhara, Takahiko Katoh

**Affiliations:** 1 Research Center for Environmental and Developmental Medical Sciences, Kyushu University, Fukuoka, Japan; 2 Department of Obstetrics and Gynecology, Kyushu University Hospital, Kyushu University, 3-1-1 Maidashi, Higashi-ku, Fukuoka, 812-8582 Japan; 3 Department of Cancer Information Research, Clinical Research Institute, National Kyushu Cancer Center, Fukuoka, Japan; 4 Department of Obstetrics and Gynecology, Graduate School of Medical Sciences, Kyushu University, Fukuoka, Japan; 5 Department of Pediatrics, Graduate School of Medical Sciences, Kyushu University, Fukuoka, Japan; 6 Japan Environment and Children’s Study, UOEH Subunit Center, University of Occupational and Environmental Health, Kitakyushu, Fukuoka Japan; 7 Department of Obstetrics and Gynecology, School of Medicine, University of Occupational and Environmental Health, Kitakyushu, Fukuoka Japan; 8 Department of Environmental Health, School of Medicine, University of Occupational and Environmental Health, Kitakyushu, Fukuoka Japan; 9 Department of Pediatrics, School of Medicine, University of Occupational and Environmental Health, Kitakyushu, Japan

**Keywords:** Maternal sleep, Small for gestational age, Birth cohort

## Abstract

**Objectives:**

Small for gestational age infants have an increased risk of immediate complications, short-term morbidity and mortality, and long-term neurologic and metabolic disorders in adulthood. Previous research has shown that reduced sleep duration is a risk factor for SGA birth. However, only a few studies have evaluated maternal sleep as a risk factor for SGA birth. In the present study, we investigated the relationship between the amount and quality of mothers’ sleep and infants’ birth weight.

**Results:**

This cohort study (n = 8631) used data from the Japan Environment and Children’s Study, an ongoing cohort study that began in January 2011. Data on sleep status (sleep duration and one indicator of sleep quality) and potential confounding factors were recorded. A log-binomial regression model was used to estimate the risk of small for gestational age birth, and the results were expressed as risk ratios and their respective 95% confidence interval. No significant results were observed for sleep duration or tiredness upon waking. Neither the amount nor the quality of mothers’ sleep was associated with the risk of small for gestational age birth.

## Introduction

Small for gestational age (SGA) infants have an increased risk of immediate complications, short-term morbidity and mortality, and long-term neurologic and metabolic disorders in adulthood [1, 2] . SGA is defined as a birth weight below the 10th percentile at any gestational age [3, 4]. Although several risk factors for SGA have been identified, some remain unknown. In our previous study, the analysis of the Japan Environment and Children’s Study (JECS) data set showed that neither severe nausea nor vomiting in early pregnancy nor hyperemesis gravidarum was associated with an increased risk for SGA birth [5].

One study showed that reduced sleep duration was a risk factor for SGA [6]. However, only a few studies have evaluated the role of maternal sleep in SGA birth. Thus, in the present study, we investigated the relationship between the amount and quality of mothers’ sleep and infants’ birth weight using data from the JECS.

## Main text

### Methods

Data used in this study were obtained from the JECS, an ongoing cohort study that was started in January 2011. The JECS was designed to follow-up mothers using a survey until their newborns reached the age of 13 years. Its objective was to elucidate the effect of environmental factors on children’s health. The detailed methodology has been previously reported [7].

In brief, pregnant women were recruited during the approximately 3-year recruitment period until March 2014. 15 study regions were selected throughout Japan. We met with as many pregnant women as possible who lived in the study area. One of the following recruitment protocols was carried out: (1) recruitment at the time of the first prenatal examination at participating health care institutions, and/or (2) recruitment at local government offices issuing the Mother–Child Health Handbook, which is a complimentary official booklet that all expecting mothers in Japan are given when they become pregnant. The JECS was conducted after obtaining written informed consent from all participants. However, we excluded those who had reasons that made it difficult for them to fill in the questionnaire in Japanese; for instance, if an individual was traveling to her hometown to deliver her baby, she could not participate in the survey [7, 8].

As of the end of 2011, 9646 participants had delivered successfully. After excluding missing data and premature birth, we analyzed the data of the remaining 8631 women who had singleton, full-term (≥37 weeks, but <42 weeks) pregnancies (Fig. 1 of the previous article [5]). The present study is based on the dataset of jecs-ag-ai-20131008, which was released in October 2013.

Follow-ups were done using self-administered questionnaires, which were filled out during the first and second trimesters of pregnancy and at 1 month after birth. We obtained the medical data by transcribing medical records which were updated during the first trimester, at the time of delivery, and 1 month after birth. The questionnaires collected data related to pregnancy history, general medical history, and confounding and modifying factors such as social and lifestyle factors. From the transcribed medical data, we collected the birth weights and other data related to pregnancy and childbirth, such as gestational age, parity, and labor complications.

The sleep index was included in the questionnaire for the second trimester. As a quantitative index of sleep, we calculated “hours of sleep” as the time interval between when a pregnant woman went to bed and got out of bed. Duration of sleep was classified into five categories based on a previous study among pregnant Japanese women [9]. As a qualitative index of sleep, we used the answer to the following question on the questionnaire based on a national health investigation [10]: “How would you rate your average mood upon waking over the previous month?” Scores of 1, 2, 3, 4, and 5 represented extremely bad, relatively bad, normal, relatively good, and extremely good, respectively. Scores of 3, 4, and 5 were used as references for 1 and 2.

The participants underwent ultrasonography during their first trimester, and for women with a difference of ≥7 days in their due date, as calculated from their last menstrual period, we used the due date derived from an ultrasound examination. Birth weights were transcribed from medical records. SGA was defined as birth weight <10th percentile of birth weight standards by gestational age for Japanese neonates [11].

The covariates of maternal age, pre-pregnancy body mass index (BMI), parity, smoking, hypertension, and alcohol consumption were included in the questionnaire for the first trimester. Covariates of education and income were included in the questionnaire for the second trimester. The covariate of maternal weight gain during pregnancy was calculated based on information from medical records.

### Statistical analyses

We assessed the relationships among hours of sleep, tiredness upon waking and SGA birth in subjects who had single, full-term births. A log-binomial regression model was used to estimate crude risk, confounder-adjusted risk and 95% confidence interval (CI) for SGA birth. The following potential factors were assessed for confounder adjusted risk: maternal age, pre-pregnancy BMI, gestational age at birth, smoking, hypertension, alcohol consumption, and education. All statistical analyses were performed using SAS version 9.3 (SAS Institute Inc., Cary, NC, USA).

## Results

The means of maternal age, gestational age at birth, and birth weight were 30.6 ± 4.98 years, 39.0 ± 1.14 weeks, and 3051 ± 369.85 g, respectively. Results of the univariate analysis for birth weight, which accounted for confounding background and social factors, are shown in a table in the previous article [5].

SGA risk ratios for participants with pre-pregnancy BMI < 18.5 kg/m^2^, who smoked, had hypertension, and had weight gain of <7 kg during pregnancy were 1.58 (95% CI 1.32–1.90), 1.48 (95% CI 1.11–1.97), 1.73 (95% CI 1.17–2.56), and 1.28 (95% CI 1.05–1.55), respectively, indicating that the risk of SGA birth was slightly elevated in these participants. When the pre-pregnancy BMI was ≥25 kg/m^2^ and weight gain during pregnancy was >12 kg, the SGA risk ratios were 0.60 (95% CI 0.43–0.85) and 0.52 (95% CI 0.41–0.66), respectively, indicating a slightly decreased risk for SGA birth.

Crude and adjusted risk ratios for the influence of sleep on birth weight are shown in Table 1. No significant results were observed for any sleep duration in our investigation. In addition, sleep quality was not associated with risk of SGA birth.

**Table 1:** Crude and adjusted risk ratios for sleep factors and birth weight

	No. of births	%	Missing data (n)	No. of non-SGA births	No. of SGA births	% SGA	Crude		Confounder-adjusted	
							RR	95% CI	RR	95% CI
Hours of sleep										
4–5.9	367	4.8	16	331	20	5.7	0.73	0.47–1.14	0.74	0.45–1.23
6–6.9	1242	16.1	68	1104	70	6.0	0.76	0.59–1.00	0.75	0.55–1.02
7–7.9^a^	2545	33.0	146	2212	187	7.8			1	
8–8.9	2205	28.6	113	1928	164	7.8	1.01	0.82–1.23	1.05	0.84–1.32
9–12	1349	17.5	74	1198	77	6.0	0.77	0.60–1.00	0.80	0.60–1.08
Missing	923		66	789	68					
Tiredness upon waking										
Bad	1970	23.3	102	1729	139	7.4	1.04	0.87–1.25	1.11	0.90–1.36
Good^a^	6492	76.7	366	5688	438	7.1			1	
Missing	169		15	145	9					

*CI* confidence interval, *RR* risk ratio, *SGA* small for gestational age

^a^Used as a reference in the calculation of RRs

## Discussion

In the present study, neither sleep duration nor tiredness upon waking was associated with risk of SGA birth. Similar to other biologic variables, daily sleep duration in any healthy adult population is normally distributed. The sleep duration of pregnant Japanese women is also normally distributed, and the most common sleep duration is 7–7.9 h [9]. According to a previous national health investigation, 20% of general adults experience bad quality of sleep [10]. Consistent with this finding, in this study, 23.3% of pregnant women experienced bad quality of sleep.

Sleep disorders during pregnancy are known to influence the occurrence of hypertension in pregnant women [12, 13]. Thus, although it was thought that sleep disorders were a risk factor for low birth weight, the results of the present study did not confirm this. Abeysena et al. [6] reported that shorter sleep duration (<8 h) was a risk factor for SGA birth when SGA was defined as a birth weight less than the fifth percentile. However, no other study has reported reduced sleep duration as a risk factor for SGA birth [14, 15]. The definition of “shorter sleep duration” may differ between countries and regions, and in some regions, time spent not sleeping may be interpreted as time at work. Thus, in future studies, it is necessary to carefully investigate the effect of various factors including sleep duration, working hours, and mental stress on the risk of SGA birth.

## Conclusions

This study showed that neither the amount nor the quality of mothers’ sleep was associated with the risk of SGA birth. Further studies will be needed to investigate the effect of various other factors such as sleep duration, working hours, and mental stress on the risk of SGA birth.

## Limitations

Our study has a methodologic limitation in that the data on sleep duration were obtained by a self-administered questionnaire, and thus, may have been prone to misclassification. However, a previous study showed that self-assessed sleep duration yielded valid results in comparison with quantitative sleep assessment using actigraphy [16]. Our study has another limitation in that although the incidence of SGA births was relatively high among mothers who lacked weight gain information, we were unable to ascertain why this was so. Mothers excluded from this study due to unavailability of weight gain information might have influenced the results.

## Abbreviations

95% CI: 95% confidence interval
BMI: body mass index
JECS: Japan Environment and Children’s Study
RR: risk ratio
SD: standard deviation
SGA: small for gestational age

## References

[1] Kok JH, den Ouden AL, Verloove-Vanhorick SP, Brand R (1998). Outcome of very preterm small for gestational age infants: the first nine years of life. Br J Obstet Gynaecol.

[2] Patterson RM, Prihoda TJ, Gibbs CE, Wood RC (1986). Analysis of birth weight percentile as a predictor of perinatal outcome. Obstet Gynecol.

[3] Committee on Practice Bulletins-Gynecology, American College of Obstetricians and Gynecologists (2001). Intrauterine growth restriction. Clinical management guidelines for obstetrician-gynecologists. Int J Gynaecol Obstet.

[4] Goldenberg RL, Cutter GR, Hoffman HJ, Foster JM, Nelson KG, Hauth JC (1989). Intrauterine growth retardation: standards for diagnosis. Am J Obstet Gynecol.

[5] Morokuma S, Shimokawa M, Kato K, Sanefuji M, Shibata E, Tsuji M (2016). Relationship between hyperemesis gravidarum and small-for-gestational-age in the Japanese population: the Japan Environment and Children’s Study (JECS). BMC Pregnancy Childbirth.

[6] Abeysena C, Jayawardana P, DE A, Seneviratne R (2009). Maternal sleep deprivation is a risk factor for small for gestational age: a cohort study. Aust N Z J Obstet Gynaecol.

[7] Kawamoto T, Nitta H, Murata K, Toda E, Tsukamoto N, Hasegawa M (2014). Working group of the epidemiological research for children’s environmental health. Rationale and study design of the Japan Environment and Children’s Study (JECS). BMC Public Health.

[8] Ministry of the Environment. Japan Environment and Children Study. 2013. http://www.env.go.jp/en/chemi/hs/jecs/. Accessed 22 Sept 2013.

[9] Suzuki K, Ohida T, Sone T, Takemura S, Yokoyama E, Miyake T (2003). An epidemiological study of sleep problems among the Japanese pregnant women. Nihon Koshu Eisei Zasshi.

[10] Ministry of Health, Labour and Welfare. National health and nutrition survey Japan. 2014. http://www.mhlw.go.jp/stf/houdou/0000106405.html**(in Japanese)**. Accessed 8 Aug 2017.

[11] Itabashi K, Miura F, Uehara R, Nakamura Y (2014). New Japanese neonatal anthropometric charts for gestational age at birth. Pediatr Int.

[12] Micheli K, Komninos I, Bagkeris E, Roumeliotaki T, Koutis A, Kogevinas M (2011). Sleep patterns in late pregnancy and risk of preterm birth and fetal growth restriction. Epidemiology.

[13] Owusu JT, Anderson FJ, Coleman J, Oppong S, Seffah JD, Aikins A (2013). Association of maternal sleep practices with pre-eclampsia, low birth weight, and stillbirth among Ghanaian women. Int J Gynaecol Obstet.

[14] Pamidi S, Pinto LM, Marc I, Benedetti A, Schwartzman K, Kimoff RJ (2014). Maternal sleep-disordered breathing and adverse pregnancy outcomes: a systematic review and metaanalysis. Am J Obstet Gynecol..

[15] Chang JJ, Pien GW, Duntley SP, Macones GA (2010). Sleep deprivation during pregnancy and maternal and fetal outcomes: is there a relationship?. Sleep Med Rev.

[16] Lockley SW, Skene DJ, Arendt J (1999). Comparison between subjective and actigraphic measurement of sleep and sleep rhythms. J Sleep Res.

